# Resolution of macular edema in Coats' disease with intravitreal bevacizumab

**DOI:** 10.4103/0301-4738.58482

**Published:** 2010

**Authors:** Morteza Entezari, Alireza Ramezani, Ladan Safavizadeh, Nader Bassirnia

**Affiliations:** Department of Opthomology, Ophthalmic Research Centre, Shahid Beheshti University (M.C.), Tehran, Iran

**Keywords:** Bevacizumab, coats' disease, macular edema

## Abstract

A 13-year-old boy was referred because of visual deterioration in his right eye. The visual acuity was two meters of counting fingers. Indirect ophthalmoscopy and biomicroscopy revealed exudative macular edema as well as tumor-like telangiectatic vessels and exudation in temporal periphery. With diagnosis of Coats' disease (stage II) confirmed by fluorescein angiography, three intravitreal injections of bevacizumab were performed at 6-week intervals. One year after the last injection, there was a significant resolution of macular edema as well as visual acuity improvement to 20/20. This is the first case report in which a distinct improvement in macular edema was observed with intravitreal bevacizumab in Coats' disease.

Coats' disease was first characterized as exudative retinopathy and retinal vascular abnormalities in 1908.[1] Coats' disease occurs in healthy young males; although females are occasionally affected. It is unilateral in 90%. The hallmark of Coats' disease is the development of telangiectasia in the retinal periphery which can lead to subretinal exudation with an affinity to the posterior pole.[2] Treatment is indicated when exudation is threatening or involves the macula. Photocoagulation and/or cryopexy have been used to treat the telangiectatic vessels; however, the visual prognosis is still not satisfactory.[3–5]

In this report, we present a patient with Coats' disease that was successfully treated with three intravitreal bevacizumab injections.

## Case Report

A 13-year-old boy was referred to our clinic with a two-month history of gradual blurring of vision in his right eye. There was no history of previous ocular disease or treatment. The patient's general health was good and the family history was unremarkable.

**Figure 1:**
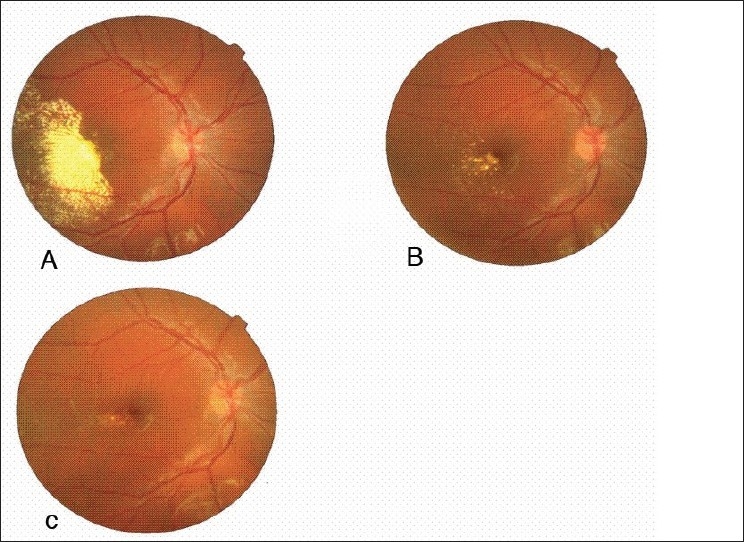
Fundus photograph (A) before injection, (B) 8 months after the third injection, (C) 12 months after the third injection

**Figure 2:**
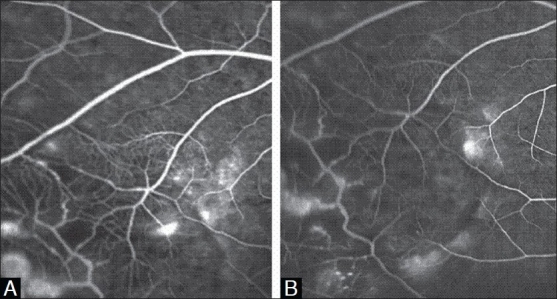
A: Baseline flourescein angiogram of the peripheral fundus showing aneurysmal dilatation (light bulb), telangiectatic vessels with arteriovenous shunts and areas of capillary non-perfusion. B: Flourescein angiogram of the peripheral fundus after 10 months of the third injection demonstrating a leakage reduction.

Right eye visual acuity was counting finger at two meters and slit lamp examination of anterior segment was normal. Fundus biomicroscopy revealed macular edema with star-shaped exudation on its temporal side [Fig. 1A]. In indirect ophthalmoscopic examination, multiple tumor-like retinal vessel abnormalities and exudation were observed in the temporal peripheral retina. The vitreous cavity was clear. Fluorescein angiograms showed leaking telangiectatic and shunt vessels as well as multiple avascular areas in the same region [Fig. 2A]. Left eye was completely normal.

With the diagnosis of stage II of Coats' disease,[6] the patient was treated by three injections of 1.25 mg (0.05 cc) intravitreal bevacizumab (Avastin, made for F. Hoffman-La Roche Ltd Basel, Switzerland by Genetech Inc., San Francisco, CA) with 6-week intervals. The injections were performed through the superotemporal quadrant, 4 mm from the limbus using 30-gauge needles. The patient was reviewed on the first postinjection days and at 1, 3, and 6 weeks after each intervention and followed up to 12 months after the last injection.

Visual acuity gradually improved to 20/60 up to 6 weeks after the first injection. While there was a significant reduction in macular exudation and edema, the telangiectatic vessels remained unchanged. Following the 2nd injection, visual acuity increased to 20/40; however, the patient complained of metamorphopsia. Fundus examination revealed more reduction in the severity of macular edema.

Three months after the 3rd injection, visual acuity improved to 20/20 and remained stable up to 12 months thereafter. At the last visit, in addition to a significant reduction of macular edema and exudation [Fig. 1B, C], the peripheral vascular lesions seemed to be regressed partially [Fig. 2B]. During the injection intervals and follow-up period up to 12 months, we did not notice any sign of relapse.

## Discussion

This case presented a dramatic response of exudative macular edema in patient with Coats' disease to intravitreal bavacizumab. The visual acuity improved from counting finger to 20/20 by three injections and remained stable for 12 months.

Several treatment modalities have been purposed for the management of Coats' disease including hormones, antibiotics, radiation, trans-scleral diathermy, photocoagulation, and cryopexy with minimal success. Recently, Jarin *et al.* demonstrated resolution of sever macular edema in adult Coats' syndrome with high-dose (25mg) intravitreal triamcinolone acetonide following an unsuccessful treatment with grid laser. However, their patient did not show any improvement in visual acuity in spite of anatomical regression of macular edema. They believed that it was likely due to the chronocity of the macular edema that once lipid plaques have deposited on the macula it is usually irreversible and often leads to permanent visual loss.[7] Nevertheless, we noticed a significant visual improvement in association with the resolution of the macular edema after intravitreal bevacizumab which might emphasize the benefit and necessity of earlier interventions in such cases before any chronic macular changes develop.

Laser photocoagulation and cryotherapy have been used for treatment of Coats' disease. They are usually directed to obliterate the abnormal telangiectatic vessels. Cryotherapy is indicted when the aneurysms are in the periphery or large areas of exudation are present beneath the telangiectatic vessels. However, one should be cautious about the complications such as premacular fibrosis which may occur as a result of excess retinal photocoagulation or cryopexy.[4] Lefaut *et al.* reported two cases with this complication following laser treatment in Coats' disease.[5] Our case presented with a large area of telangiectatic vessels with severe exudative macular edema which required a heavy conventional treatment with either laser or cryotherapy. We preferred to use intravitreal bevacizumab instead to avoid such side effects. No complication was encountered in this case.

Standard treatment of Coats' disease includes laser or cryo retinopexy to the telangiectatic vessels and anti-VEGF injections seems to be an adjunctive treatment for these methods.[4] Nonetheless, one concern always exists regarding the temporary effect of the drugs given by intravitreal route without any accompaning laser or cryotherapy. However, we noticed sclerotic changes developed in the abnormal vessels which might indicate a more permanent effect of this therapy. Using multiple injections might help us to achieve this result.

To the best of our knowledge, this is the first report of successful treatment of macular edema in Coats' disease with intravitreal bevacizumab. Nonetheless, no practicable conclusion could be drawn from this report and a randomized clinical trial is mandatory to prove the efficacy and safety of this treatment modality.

## References

[1] Coats G (1908). Forms of retinal disease with massive exudation. Roy London Ophthalmol Hospital Report.

[2] Sanborn GE, Magargal LE, Jaeger EA (2004). Venous occlusive disease of the retina. Duane's clinical ophthalmology [book on CD-ROM].

[3] Kolar P, Vikova E, Lieber S (2003). Milliary aneurysm. Cerk Slov Ophthalmol.

[4] Sugimoto M, Sasoh M, Ito Y, Miyamura M, Uji Y, Chujo S (2002). A case of Coats' disease with peeling of premacular fibrosis after photocoagulation. Acta Ophthalmol Scand.

[5] Lefaut BA, Priem H, De Laey JJ (1996). Premacular fibrosis in juvenile Coats' disease with spontaneous peeling after photocoagulation of the congenital vascular anomalies. Ball Soc Belge Ophthalmol.

[6] Shields JA, Shields CL, Honavar SG, Demirci H, Cater J (2001). Classification and management of Coats' disease: The 2000 Protocor lecture. Am J Ophthalmol.

[7] Jarin RR, Teoh SC, Lim TH (2006). Resolution of severe macular oedema in adult Coats' syndrome with high-dose intravitreal triamcinolone acetonide. Eye.

